# GC-MS analysis, anti-inflammatory and anti-proliferative properties of the aerial parts of three *Mesembryanthemum spp.*

**DOI:** 10.1016/j.toxrep.2024.101829

**Published:** 2024-11-28

**Authors:** Heba R. Mohamed, Manal M. Hamed, Eman A. El-Wakil, Hend Okasha

**Affiliations:** a Department of Medicinal Chemistry, Theodor Bilharz Research Institute, Kornish El-Nile, Warrak El-Hadar, Giza 12411, Egypt; b Department of Biochemistry and Molecular Biology, Theodor Bilharz Research Institute, Kornish El-Nile, Warrak El-Hadar, Giza 12411, Egypt

**Keywords:** *Mesembryanthemum* spp., GC-MS, anti-inflammatory, RBCs toxicity, HCC, Colon cancer

## Abstract

**Background:**

Due to their variability and safety, widespread research on phytochemicals continually encourages researchers to study various plants for their potential health benefits.

**Objectives:**

This study aims to explore the phytochemical constituents of the aerial parts of three *Mesembryanthemum* spp.; *M. nodiflorum*, *M. forsskaolii,* and *M. cordifolium* existed in Egyptian nature using GC-MS analysis and studying their different biological activities in correlation to computational analysis.

**Methods:**

Investigation of in vitro anti-inflammatory and anticancer activities and in silico studies of identified major compounds on VEGFR. Results: Thirty-three compounds were identified, octadecanoic acid, 2, 3-dihydroxypropyl ester, and 1H-Indene, 1-hexadecyl-2, 3-dihydro are the common compounds in the three extracts with different percentages. *M. forsskaolii* is the most extract with diverse phytoconstituents showing significant anticancer properties against the CACO2 cells with IC_50_ value equal to 31.78 µg/mL. Nevertheless, all extracts showed potent anti-inflammatory activity at high concentrations (500 µg/mL). *M. nodiflorum*, *M. forsskaolii*, and *M. cordifolium* had IC_50_ on HepG2 cells equal to 73.64, 88.18, and 87.82 µg/mL. Molecular findings showed the three extracts had distinct effects on apoptosis modulation in HepG2 cells. Conclusion The findings suggest that the studied extracts had potential therapeutic properties as anti-inflammatory and anticancer agents, supported by an in-silico interaction study.

## Highlights

• Thirty-three compounds identified of three *Mesembryanthemum* species extracts,
• *M. forsskaolii* exhibited the highest diversity in phytoconstituents.
• *M. forsskaolii*'s anticancer activity against CACO2 cells with IC_50_ of 31.78 µg/mL, and IC_50_ for HepG2 cells were 73.64, 88.18, and 87.82 µg/mL for *M. nodiflorum*, *M. forsskaolii*, and *M. cordifolium*, respectively.
• Molecular studies highlighted apoptotic modulation effects on HepG2 cells of the three extracts.

## Graphical Abstract

**Figure:**
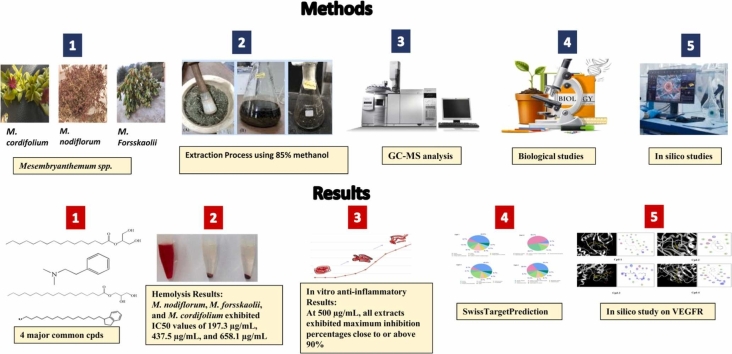

## 1 Introduction

Chemical compounds obtained in pharmaceutical studies from medicinal plant extractions are used in complementary medicine because of their variable pharmacological properties [1]. Mutually, natural extracts and purified compounds are used in traditional medicine in many countries more than synthetic drugs due to their safety, availability, and low-cost benefits.

Natural products contain enormous compound phytochemicals capable of their therapeutic properties, such as polyphenols, carotenoids, phytosterols, and polysaccharides. Seo and Ju suggest that Mesembryanthemum crystallinum L. (ice plant) possesses antioxidant and growth-inhibitory activities in colon cancer cells [2].

Cancer is a major health problem worldwide, caused by uncontrolled cell growth, and is a disease highly associated with several genetic disorders, lifestyle, nutrition, and environmental factors [3], [4]. Cancer treatments include radiotherapy, chemotherapy, and surgery mostly followed by pharmacotherapy [5]. However, in many cases, these approaches have some drawbacks that affect a patient’s life [6]. Till now, natural products and alternative medicine have entered into radio-diagnostic and pharmacoproteomic modalities to improve treatment [7]. In cancer therapy many drugs of plant origin are utilized such as Paclitaxel; from *Taxus brevifolia*, which was FDA-approved in the treatment of breast, ovarian, and lung cancer, as well as Kaposi's sarcoma, Vincristine; from *Catharanthus roseus*, was FDA approved in treatment of leukemia, lymphoma, and solid tumors, and Irinotecan; from *Camptotheca acuminata*, was FDA approved in treatment of colorectal cancer and other solid tumors [8], [9], [10].

One major driver for cancer angiogenic switch is the vascular endothelial growth factor receptor (VEGFR) and its signaling pathway, which usually deregulated many cancer types including hepatocellular carcinoma (HCC) and colon cancer [11]. Therefore, blockage of VEGFR in cancer cells through binding with a specific chemical compound causes tumor starvation in addition to low oxygen supplementation. FDA-approved sorafenib, sunitinib, and axitinib are considered small-molecule inhibitors of the VEGFR [12], [13]. The programmed cell death mechanism; apoptosis, is the major regulator in cellular homeostasis, thus suppressing carcinogenesis [14]. Dysregulation of the apoptotic routes can be a favoring factor in the pathological proliferation of malignant cells and cancer progression. Angiogenesis is started through VEGFR which prevents apoptosis in endothelial cells [15]. This anti-apoptotic effect is also useful for the maintenance and increase of endothelial cells, which should be preserved for the creation of new blood vessels for cancer cells [16]. However, the role of the VEGFR signaling pathway is not only an induction of cell proliferation but it can lead to the inhibition of apoptosis in some cancer cells causing their uncontrollable growth [17], [18].

This study examined the anti-inflammatory and anticancer activities of methanol extracts of the aerial parts of *M. nodiflorum*, *M. forsskaolii,* and *M. cordifolium* in addition to the determination of their GC-MS constituents via GC-MS to identify the most dominant compounds in the three extracts for *in silico* study on VEGFR as a receptor target. Another computational analysis using SwissTargetPrediction to identify the targets facilitates the understanding of the biological effects of compounds in various research contexts.

## 2 Methods

### 2.1 Plant material

The aerial parts of three *Mesembryanthemum* spp. (F: Aizoaceae); *Mesembryanthemum nodiflorum* L.*, M. forsskaolii* Hochst. Ex Boiss and *M. cordifolium* L.f., presented along the Cairo-Alexandria desert road, Egypt, were collected and authenticated with the assistance of Prof. Dr. Rim Hamdy, professor of plant taxonomy and flora, Faculty of Science, Cairo University. Voucher specimens were kept at the herbarium of Cairo University. The three understudied plants were allowed to dry in a shaded place at ambient temperature for nearly three weeks then ground into fine powder and finally kept in dark air-tight bags till extraction [19], [20].

### 2.2 Extraction process

The crude extracts of the aerial parts of the selected *Mesembryanthemum* species were prepared through the maceration technique via soaking the pre-prepared fine powders (50–70 gm) in 85 % methanol for five days at room temperature with intermittent shaking. The three extracts were then filtered and concentrated at reduced pressure and temperature using a rotary evaporator (BUCHI, Germany). This process was repeated three times for each extract affording three crude extracts of uniform weight. The three crude extracts were then preserved in dark and clean veils for further investigation [21].

### 2.3 GC-MS analysis

GC-MS analysis of the three crude extracts was conducted through Shimadzu QP2020 GC-MS equipped with GC-2010 plus (Kyoto, Japan) provided with a DB-1MS fused silica column (100 % polydimethylsiloxane, 30 m×0.25 mm, 25*μ*m film thickness) and a split–splitless injector. The initial temperature of the column oven was adjusted at 50 °C, isothermal for 3 min, then increased to 300 °C with a rate of 5 °C/min and maintained at 300 °C for 10 min. The total run time was 63 min. The ion source temperature was 220 °C and the interface temperature was 280 °C. Two µL of each sample diluted with hexane and dichloromethane were injected into the GC apparatus, and the injector temperature was set to 280 °C with a split ratio of 1: 5. The carrier gas (Helium) flow rate was 1.37 mL/min with 80.00 kPa pressure; ionization voltage of 70 eV; detector voltage of 0.84 kV; scan interval of 0.3 seconds; scan speed of 1666; scan range from 35 to 500 amu. Data acquisition was performed using Lab-Solution GC-MS version 4.45SP1 Software. The identification of the separated phytoconstituents occurred by comparing their mass spectra with those of known compounds stored in the database of the National Institute of Standard and Technology (NIST 2017) as well as literature and hence the name, molecular weight and structure of these compounds were determined. The relative % of each compound was calculated by comparing its average peak area to the total areas.

### 2.4 Evaluation of cytotoxicity using RBCs hemolysis assay

A blood sample from a healthy female volunteer was used to isolate RBCs according to the guidelines of our Institutional Ethical Committee. RBCs pellet was obtained according to Okasha et al. method [22]. RBCs suspension was exposed to differing concentrations dissolved in 1x PBS, pH 7.4 (62.5, 125, 250, 500, and 1000 µg/mL) and Diclofenac was used as a reference drug. To evaluate the level of hemolysis, percentage (%) was estimated using the ratio of the absorbance of the samples against the negative and positive controls. Each sample was repeated three times to establish the significance of variance between the examined samples [23].

### 2.5 Anti-inflammatory activity using protein denaturation

Using bovine serum albumin as a protein model, the extracts and reference drug (diclofenac sodium), were dissolved in 1x PBS, pH6.4. Various concentrations of the tested extracts that had low hemolysis percent according to results obtained from RBCs hemolysis assay were prepared (200, 100, 50, 25, and 12.5 µg/mL) according to Rashdan et al [24]. The percentage inhibition of protein denaturation was determined using the following formula:

% of inhibition = (Abs_C_ - Abs_T_ / Abs_C_) x 100

Abs_C_: Absorbance of control, Abs_T_: Absorbance of tested sample

Each sample was performed in triplicate, and the results were expressed as mean ± standard deviation.

### 2.6 In silico studies of the dominant compounds in the three plants’ extracts

The potential anticancer properties of the dominant compounds in all extracts were evaluated in silico on a specific receptor related to HCC. To perform this, the following steps were established:

#### 2.6.1 Ligand preparation

The chemical compounds of interest, as going to be described through SMILES. These ligands were drawn and optimized using cheminformatics tools to present their correct stereochemistry, protonation state, and geometry. The structure of each compound was then obtained in a three-dimensional (3D) format and further optimized for formulating the final result analysis.

#### 2.6.2 Target prediction

Regarding the identification of protein-ligand interaction, SwissTargetPrediction (http://www.swisstargetprediction.ch/), Binding DB (https://www.bindingdb.org/rwd/bind/index.jsp), and SwissDock (https://swissdock.ch/) online servers were used. These tools are based on both two-dimensional and three-dimensional similarity indices to identify potential protein targets and their probable bioactivities. The ligand structure (SMILES format) and the protein of interest in PDB format were uploaded and the output was assessed according to their potential interactions [25].

### 2.7 Anticancer activity on HepG2 and CACO2 cell lines

Both HepG2 and CACO2 cell lines were purchased from VACSERA, Egypt. Using RPMI media (10 % FBS, 1 % antibiotic antimycotic, 1 % HEPES) obtained from Biobasic Co., South Korea. The cells were seeded at a density of 7000 cells/ well in 96-well plates for 24 h in a 5 % CO_2_ incubator at 37 °C to form a confluent sheet. Then, cells were exposed to different extract concentrations (200, 100, 50, 25, and 12.5 µg/mL) and DOX was used as a reference drug. The plate was incubated for 24 h, cytotoxicity was assessed using crystal violet assay as previously described [22]. The results were expressed as the relative percentage of absorbance compared to the control. The experiments were done in triplicates, and the half-maximal inhibitory concentration (IC_50_) was calculated using GraphPad Prism software.

### 2.8 Detection of apoptotic markers in treated HepG2 cells

Evaluation of apoptotic induction for each methanol extract was performed by relative quantification of oncogenes BCL-2 and AFP, and apoptotic genes BAX, BID, and CAS-3, and PUMA (p53 upregulated modulator of apoptosis) in a 24-well tissue culture plate containing 1×10^5^ HepG2 cells/well. After adding the methanol extract of each plant (dissolved maintenance RPMI media containing 2 % FBS, 1 % antibiotic antimycotic, and 1 % HEPES) at a concentration below its IC_50_, the plate was incubated for 24 h in a 5 % CO_2_ incubator at 37°C. Cells were harvested for total RNA isolation using RNA extraction kit according to the manufacturer’s instruction (Thermo Fisher Scientific, USA). The quantification of relative expression of genes of interest was performed by using Novo™ cDNA Kit (Biovision, Inc) for the synthesis of the cDNA, followed by qPCR using Maxima SYBR Green master mix (Thermo Fisher Scientific, USA). The cycling parameters were: denaturation at 95°C for 15 min and then repeated 50 cycles of denaturation at 95°C for 20 s, annealing at the specific temperature for each primer (Biovision, Inc, USA); Table 1, and extension at 72°C for 25 s. The relative expression of each detected marker was calculated and represented by the mean of the triplicate of each reaction. The expression levels of the gene of interest were determined using the formula 2^-ΔΔct [14], [26].

**Table 1:** Primers used in qPCR on HepG2 cells.

**Name**	**Sequence**	**Annealing Temperature**	**Reference**	**Accession No.**
**BCL−2**	GATGTGATGCCTCTGCGAAG	52 °C	[27]	XM_047437733.1
	CATGCTGATGTCTCTGGAATCT			
**BAX**	CCCGAGAGGTCTTTTTCCGAG	55 °C	[28]	XM_047439168.1
	CCAGCCCATGATGGTTCTGAT			
**BID**	CCTTGCTCCGTGATGTCTTTC	54 °C	[29]	NM_197966.3
	GTAGGTGCGTAGGTTCTGGT			
**CAS−3**	AGAGTCTGTGCCCAAATCAAC	50 °C	[30]	XM_054350958.1
	GCTGCTTCTCTCTTTGCTGAA			
**PUMA**	GGAGGGTCCTGTACAATCTC	51 °C	[31]	NM_001134.3
	GTGCAGGCACCTAATTGGG			
**GAPDH**	ATTCCACCCATGGCAAATTC	52 °C	[26]	NR_152150.2
	AGCATCGCCCCACTTGATT			

### 2.9 Detection of α-fetoprotein and lactate dehydrogenase in treated HepG2 cells

#### 2.9.1 α-fetoprotein (AFP)

Using sandwich ELISA (BT LAB, Cat. No. BPE245) human α-fetoprotein (AFP) was detected in the treated HepG2 cells with different extracts compared to untreated cells. In a 24-well tissue culture plate containing 1×10^5^ HepG2 cells/well, the methanol extract of each plant (dissolved maintenance RPMI media) at a concentration below its IC_50_, the plate was incubated for 24 h in a 5 % CO2 incubator at 37°C. Media were then collected for lactate dehydrogenase detection and cells were collected in 500 µL of 100 mM Tris HCl pH 7.6. According to kit instructions, 25 µL of cell lysate were applied to the pre-coated ELISA plate. The plate was read using an ELISA reader at 450 nm. Each sample was repeated three times and mean and SD were calculated.

#### 2.9.2 Lactate dehydrogenase (LDH)

LDH was detected in the collected media using LDH kit (Spectrum, Cat. No. 283005) according to the kit instructions. In brief, media samples were taken from treated HepG2 cells and subjected to centrifugation to precipitate any cell debris out of the solution. The absorbance was then taken at the indicated wavelength (340 nm) and the activity of LDH was quantified in U/L.

## 3 Results

### 3.1 GC-MS analysis

**Fig. 1:**
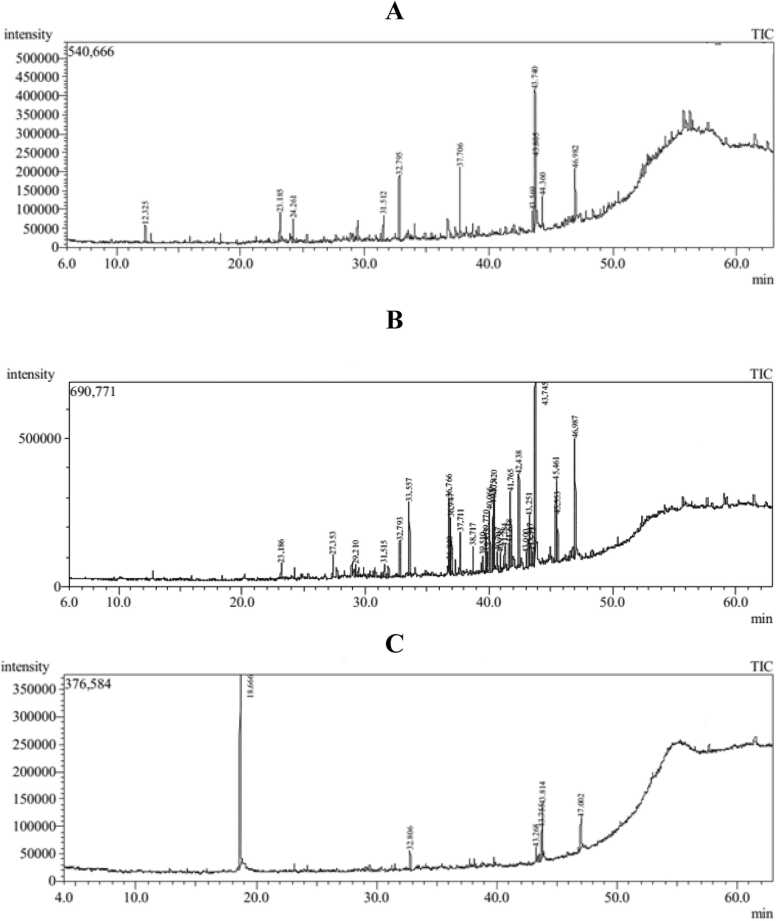
**:**GC-MS chromatograms of the aerial parts’ extracts of three *Mesembryanthemum spp**.*** A: TIC of *M. nodiflorum*; B: TIC of *M. forsskaolii*; C: TIC of *M. cordifolium*.

GC-MS analysis of the crude methanol extracts of the aerial parts of *M. nodiflorum*, *M. forsskaolii,* and *M. cordifolium* revealed the identification of thirty-three compounds constituting 99.91 %, 96.66 %, and 97.08 %, respectively of the total composition of each extract. These compounds represented in Table 2 were interpreted by comparing their mass spectra and retention times with published data and library searches. The GC-MS chromatograms of the three extracts are displayed in Fig. 1. Ten out of twelve compounds were identified from *M. nodiflorum* crude extract. Hexadecanoic acid, 2-hydroxy-1-(hydroxymethyl) ethyl ester (2-Palmitoylglycerol) (19.22 %), 1H-Indene, 1-hexadecyl-2, 3-dihydro (16.02 %) and Octadecanoic acid, 2, 3-dihydroxypropyl ester (glyceryl monostearate) (13.20 %) represented the major phytoconstituents in *M. nodiflorum*. Meanwhile, six out of eight compounds were identified from *M. cordifolium* where benzeneethanamine, N, N-dimethyl- is the dominant compound representing 46.20 % of the total extract composition followed by 1H-Indene, 1-hexadecyl-2, 3-dihydro (14.58 %) and Octadecanoic acid, 2, 3-dihydroxypropyl ester (glyceryl monostearate) (14.19 %). Also, twenty-nine out of thirty-three compounds were identified from *M. forsskaolii* and hence it is considered the richest extract among the three studied extracts with phytoconstituents. Hexadecanoic acid, 2-hydroxy-1-(hydroxymethyl) ethyl ester (2-Palmitoylglycerol) (13.93 %) and Octadecanoic acid, 2, 3-dihydroxypropyl ester (glyceryl monostearate) (9.87 %) are the major components in *M. forsskaolii* extract. Octadecanoic acid, 2,3-dihydroxypropyl ester (glyceryl monostearate), and 1H-Indene, 1-hexadecyl-2, 3-dihydro are the common compounds in the three extracts with different percentages while hexadecanoic acid, 2-hydroxy-1-(hydroxymethyl)ethyl ester is the common compound in both *M. nodiflorum* and *M. forsskaolii* only. The chemical structure, the type, the mass spectra, and the reported biological activity if found of the most abundant phytoconstituents in the three understudied extracts were displayed in Table 3.

**Table 2:** GC-MS analysis of the aerial parts’ extracts of three *Mesembryanthemum spp.*

**Peak no.**	**Rt.**	**Compound**	**Area %**			**Molecular Weight**	**Molecular Formula**	**Chemical Structure**
			*M. nodiflorum*	*M. forsskaolii*	*M. cordifolium*			
1	12.33	Isophorone	5.22	–––	–––	138	C_9_H_14_O	
2	18.67	Benzeneethanamine, N,N-dimethyl-	–––	–––	**46.20**	149	C_10_H_15_N	
3	23.19	1s,4R,7R,11R−1,3,4,7-Tetramethyltricyclo[5.3.1.0(4,11)]undec−2-en−8-one	6.50	1.15	–––	218	C_15_H_22_O	
4	24.26	2,4-dimethyl-eicosane	5.61	–––	–––	310	C_22_H_46_	
5	27.35	7-Oxabicyclo[4.1.0]heptan−3-ol, 6-(3-hydroxy−1-butenyl)−1,5,5-trimethyl	–––	1.77	–––	226	C_13_H_22_O_3_	
6	29.21	Methyl myristate	–––	0.91	–––	242	C_15_H_30_O_2_	
7	31.52	2-(2-Diethylamino-ethoxy)-fluoren−9-one	6.37	0.99	–––	295	C_19_H_21_NO_2_	
8	32.80	7,9-Di-tert-butyl−1-oxaspiro(4,5)deca−6,9-diene−2,8-dione	10.05	2.29	6.80	276	C_17_H_24_O_3_	
9	33.56	Hexadecanoic acid, methyl ester (Methyl palmitate)	–––	4.14	–––	270	C_17_H_34_O_2_	
10	36.77	9,12-Octadecadien−1-ol, (*Z*,*Z*)-	–––	4.20	–––	266	C_18_H_34_O	
11	36.84	9,12,15-Octadecatrienoic acid, methyl ester, (Z,Z,Z)- (Methyl linolenate)	–––	0.97	–––	292	C_19_H_32_O_2_	
12	36.95	9-Octadecenoic acid, methyl ester, (*E*)- (Methyl elaidate)	–––	3.36	–––	296	C_19_H_36_O_2_	
13	37.71	l-Norvaline, N-(2-methoxyethoxycarbonyl)-, hexyl ester	11.16	2.59	–––	303	C_15_H_29_NO_5_	
14	38.72	Tridecanedioic acid, dimethyl ester	–––	2.41	–––	272	C_15_H_28_O_4_	
15	39.51	Hexadecanedioic acid, dimethyl ester	–––	1.20	–––	314	C_18_H_34_O_4_	
	43.27				5.69			
16	39.77	Pentadecanoic acid, 14-oxo-, methyl ester	–––	2.66	–––	270	C_16_H_30_O_3_	
17	39.88	Hexadecanoic acid, 2-hydroxy−1-(hydroxymethyl)ethyl ester (2-Palmitoylglycerol)	–––	2.10	–––	330	C_19_H_38_O_4_	
18	40.07	9-t-Butyltricyclo[4.2.1.1(2,5)]decane−9,10-diol	–––	5.11	–––	224	C_14_H_24_O_2_	
	40.38			4.37				
19	40.42	Nonanoic acid, 9-oxo-, methyl ester	–––	3.96	–––	186	C_10_H_18_O_3_	
20	40.71	Bicyclo[3.1.1]heptan−2-ol, 2,6,6-trimethyl-	–––	1.59	–––	154	C_10_H_18_O	
21	40.96	7-Hexadecenal, (*Z*)-	–––	1.45	–––	238	C_16_H_30_O	
22	41.28	4,8,12,16-Tetramethylheptadecan−4-olide	–––	1.51	–––	324	C_21_H_40_O_2_	
23	41.66	6-Nonenal, 3,7-dimethyl-	–––	1.93	–––	168	C_11_H_20_O	
24	41.77	Undec−10-ynoic acid, undec−2-en−1-yl ester	–––	4.87	–––	334	C_22_H_38_O_2_	
25	42.44	Tricyclo[20.8.0.0(7,16)]triacontane, 1(22),7(16)-diepoxy-	–––	**5.29**	–––	444	C_30_H_52_O_2_	
26	43.00	Tetradecanedioic acid, dimethyl ester	–––	1.08	–––	286	C_16_H_30_O_4_	
27	43.25	Eicosanedioic acid	–––	3.45	–––	342	C_20_H_38_O_4_	
28	43.35	Octadecanoic acid, 2-hydroxy−1-(hydroxymethyl)ethyl ester	–––	1.55	–––	358	C_21_H_42_O_4_	
29	43.57	**1H-Indene, 1-hexadecyl−2,3-dihydro**	**5.92**	1.11	–––	342	C_25_H_42_	
	43.81		**10.10**	–––	**14.58**			
30	43.75	Hexadecanoic acid, 2-hydroxy−1-(hydroxymethyl)ethyl ester (2-Palmitoylglycerol)	**19.22**	**13.93**	9.62	330	C_19_H_38_O_4_	
31	44.36	Phthalic acid, di(2-propylpentyl) ester	6.56	–––		390	C_24_H_38_O_4_	
32	45.46	Oxiraneoctanoic acid, 3-octyl-, methyl ester, cis-	–––	4.85	–––	312	C_19_H_36_O_3_	
33	47.00	**Octadecanoic acid, 2,3-dihydroxypropyl ester (glyceryl monostearate)**	**13.20**	**9.87**	**14.19**	358	C_21_H_42_O_4_	
**% of identified compounds**			**99.91**	**96.66**	**97.08**			
**% of un-identified compounds**			**0.09**	**3.34**	**2.92**

**Table 3:** MS spectra, Chemical structure, and biological activity of the most abundant compounds in the three extracts.

**Name**	**Type**	**Species**	**Hit spectrum**	**Biological activity**
Benzeneethanamine, N,N-dimethyl-	Primary amine	*M. cordifolium*		No activity reported
Hexadecanoic acid, 2-hydroxy−1-(hydroxymethyl)ethyl ester (2-Palmitoylglycerol)	monoglyceride	*M. nodiflorum* *M. forsskaolii*		Antioxidant, antiandrogenic, hemolytic, hypocholesterolemic, nematicide
1H-Indene, 1-hexadecyl−2,3-dihydro	Bicyclic	*M. nodiflorum* *M. forsskaolii* *M. cordifolium*		Antioxidant
Octadecanoic acid, 2,3-dihydroxypropyl ester (glyceryl monostearate)	monoglyceride	*M. nodiflorum* *M. forsskaolii* *M. cordifolium*		No activity reported

### 3.2 Evaluation of cytotoxicity using RBCs hemolysis assay

**Fig. 2:**
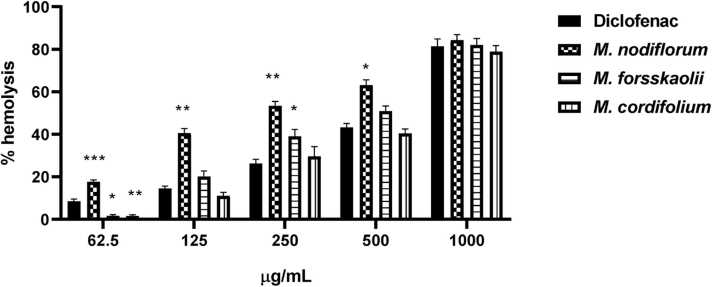
Hemolysis percent of different concentrations of *M. nodiflorum*, *M. forsskaolii*, and *M. cordifolium* extracts compared to Diclofenac as a standard drug. (*p*-value= 0. 018).

The 50 % hemolysis for Diclofenac and three different extracts were analyzed. Diclofenac revealed an IC_50_ of 593.4 µg/mL, however, extracts of *M. nodiflorum*, *M. forsskaolii*, and *M. cordifolium* exhibited IC_50_ values of 197.3 µg/mL, 437.5 µg/mL, and 658.1 µg/mL, respectively. Furthermore, statistical analysis using two-way ANOVA revealed a significant difference between the groups with *p*-value equals 0. 018. As presented in Fig. 2 a bar chart outlined the comparison between Diclofenac as a standard drug with the extracts. Results obtained showed that *M. cordifolium* extract was safer than Diclofenac followed by *M. forsskaolii* extract and the most toxic extract was that of *M. nodiflorum*.

### 3.3 In vitro anti-inflammatory activity of plants’ extracts

**Fig. 3:**
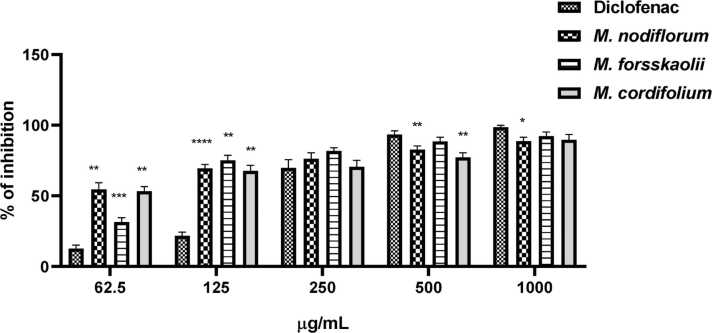
Percent inhibition of protein denaturation at different concentrations (62.5, 125, 250, 500, and 1000 µg/mL) for Diclofenac and extracts of *M. nodiflorum*, *M. forsskaolii*, and *M. cordifolium*. Statistical significance is indicated by asterisks: ***p < 0. 01, ***p < 0. 001, ****p < 0. 0001.

Results of protein inhibition percent exhibited that all tested extracts had anti-inflammatory activity. As shown in Fig. 3, the percent of inhibition for Diclofenac, and extracts of *M. nodiflorum*, *M. forsskaolii*, and *M. cordifolium* was increased with concentration demonstrating a dose-depend manner. In concentrations higher than 250 µg/mL, all the tested samples including Diclofenac showed similar and marginally different percentages of inhibition. At a concentration equivalent to 500 µg/mL, all samples exhibited maximum inhibition percentages close to or above 90 % indicating maximum anti-inflammatory response and this concentration is almost considered a plateau value.

### 3.4 Computational studies related to HCC on dominant compounds in extracts from three plants

#### 3.4.1 Target prediction

**Fig. 4:**
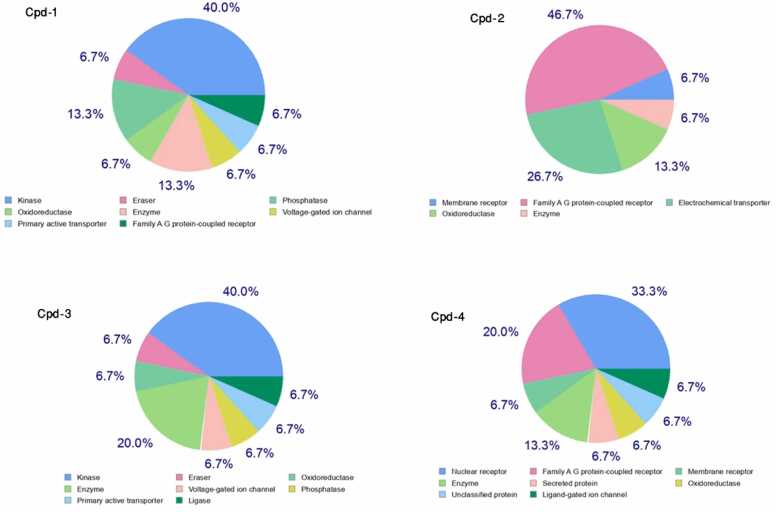
Predicted Targets from SwissTargetPrediction. The pie chart illustrates the distribution of predicted targets classified by protein type of the four dominant compounds.

The GC-MS analysis identified the dominant compounds in the extracts from three plants, *M. nodiflorum*, *M. forsskaolii*, and *M. cordifolium* as depicted in Table 4. The SwissTargetPrediction analysis results in Fig. 4 showed that For Cpd-1, the main hit went to kinases (40 %) followed by oxidoreductases and enzymes with 13.3 % each indicating the compound’s potential in phosphorylation and redox reactions. Cpd-2 mainly acts on family A G protein-coupled receptors (46.7 %), oxidoreductase (26.7 %), and enzyme (13.3 %), suggesting that it has effects on signal transduction. It is seen that only 40 % of the targets for Cpd-3 are kinases and 20 % are enzymes, which suggests that Cpd-3 may modulate the phosphorylation and enzymatic processes. The molecular targets of Cpd-4 are diverse, nuclear receptors are targeted most significantly (20 %) followed by family A G protein-coupled receptors (33. 3 %) and membrane receptors (20 %), this indicates its interaction with intracellular and membrane protein suggesting gene regulation and signals transduction.

**Table 4:** dominant compound in the three plants’ extracts according to GC-MS analysis.

**Plant**	**Dominant Compound**	**Key**	**Structure**
***M. nodiflorum*** ***M. forsskaolii***	Hexadecanoic acid, 2-hydroxy−1-(hydroxymethyl)ethyl ester	Cpd−1	CCCCCCCCCCCCCCCC(=O)OCC(CO)O
***M. cordifolium***	Benzeneethanamine, N,N-dimethyl	Cpd−2	CN(C)CCc1ccccc1
***M. nodiflorum*** ***M. forsskaolii*** ***M. cordifolium***	Octadecanoic acid, 2,3-dihydroxypropyl ester	Cpd−3	CCCCCCCCCCCCCCCCCC(=O)OCC(CO)O
	1H-indene, 1-hexadecyl−2,3-dihydro	Cpd−4	CCCCCCCCCCCCCCCCC1CCC2=CC=CC=C12

#### 3.4.2 Docking study on VEGFR

**Fig. 5:**
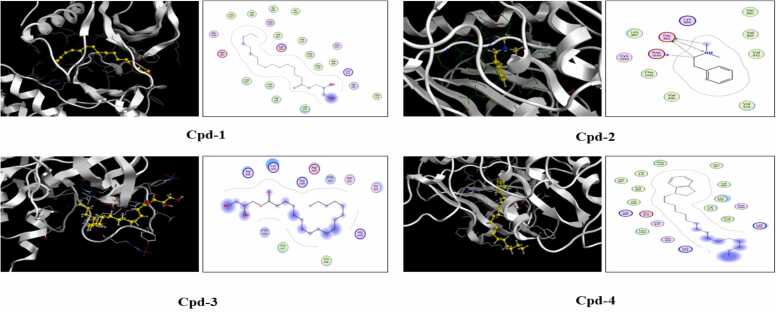
Molecular docking of the four major compounds and VEGFR. The docking poses and the binding interactions of each compound with VEGFR are shown in the various panels. The yellow color represents the compound molecule in 2D and the white color represents the receptor; VEGFR.

Results showed that cpd-4 had the highest binding affinity equal to −8.185 kcal/mol. In addition, Cpd-1 showed a high binding affinity that equals −7.739 kcal/mol. However, Cpd-2 had a slightly low binding affinity of −5.299 kcal/mol. Cpd-3 exhibited a middle rank at the docking score of −6.721 kcal/mol. These results also show that the four compounds have different levels of binding affinity, which could consequently affect the levels of modulation of VEGFR signaling and subsequent angiogenesis. Fig. 5

### 3.5 Anticancer activity of the three plants’ extracts on CACO2 and HepG2 cell lines

**Fig. 6:**
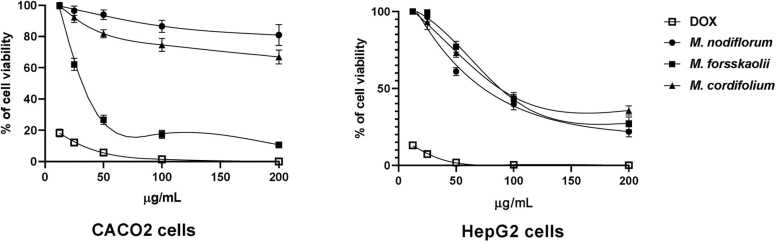
Results of the cell viability test based on varying concentrations of *M. nodiflorum*, *M. forsskaolii*, and *M. cordifolium* extracts compared to DOX as a reference drug. The line graph is a depiction of the extracts' effect on the Cell viability of both CACO2 and HepG2 cells. The *M. forsskaolii* extract showed a remarked viability decrease even with a lower concentration of the extract on CACO2 cells with IC_50_ equal to 31.78 µg/mL. However, *M. nodiflorum*, and *M. cordifolium* extracts showed no observed IC_50_. In HepG2 cells, the three extracts showed a closure calculated IC_50_ values ranging from 73.64 to 88.18 µg/mL.

**Fig. 7:**
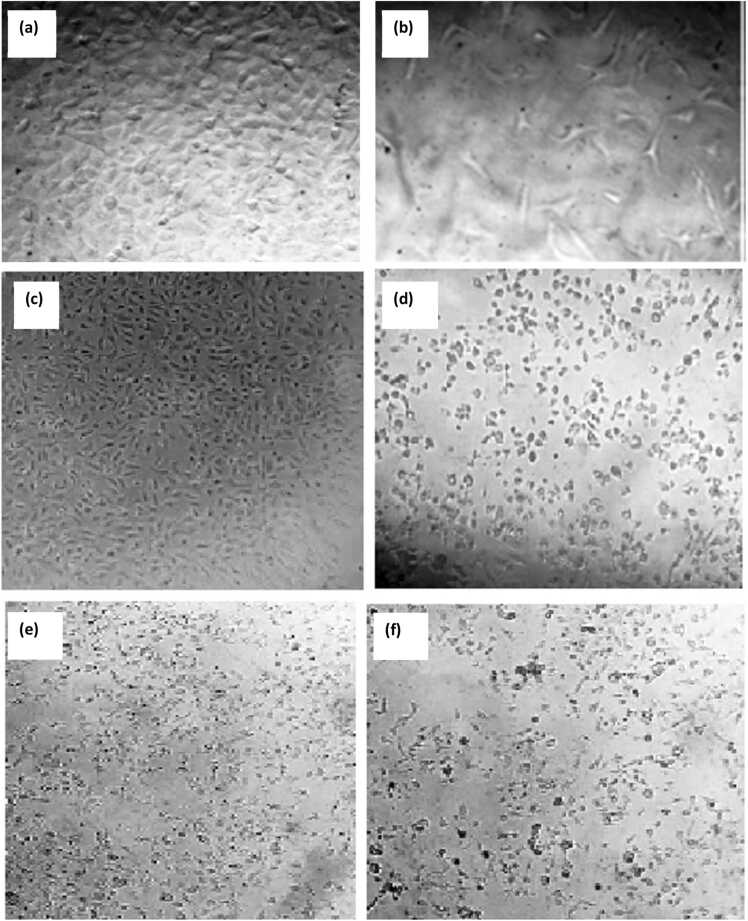
Microscopic examination of HepG2 cells subjected to different extracts compared to untreated cells. The untreated cells had a confluent sheet however treated cells showed morphological changes including cell shrinking, decreased round size, and detached cells. (a): Untreated cells CACO2 cells, (b): Treated cells with *M. forsskaolii* at a concentration of 25 µg/mL, (c): Untreated HepG2 cells, (d): Treated cells with *M. nodiflorum,* (e): Treated cells with *M. forsskaolii*, and (f): Treated cells with *M. cordifolium* at a concentration equivalent to 100 µg/mL.

Results of anticancer activity of extracts of *M. nodiflorum*, *M. forsskaolii*, and *M. cordifolium* on CACO2 cells at different concentrations showed that *M. nodiflorum* extract and *M. cordifolium* extract had no observed IC_50_ at the tested concentrations. However, *M. forsskaolii* extract showed a remarked anticancer activity on CACO2 cells with calculated IC_50_ equals 31.78 µg/mL. Nevertheless, the extracts exhibited a significant anticancer activity on HepG2 cells where *M. nodiflorum*, *M. forsskaolii*, and *M. cordifolium* had calculated IC_50_ equals 73.64, 88.18, and 87.82 µg/mL**,** respectively. Fig. 6. Microscopic examination of treated cells (CACO2 and HepG2) with the extracts showed cell morphological changes such as round cells with shrinking sizes and decreased numbers compared to untreated cell control **(**Fig. 7**)**.

### 3.6 Detection of apoptotic markers in treated HepG2 cells

**Fig. 8:**
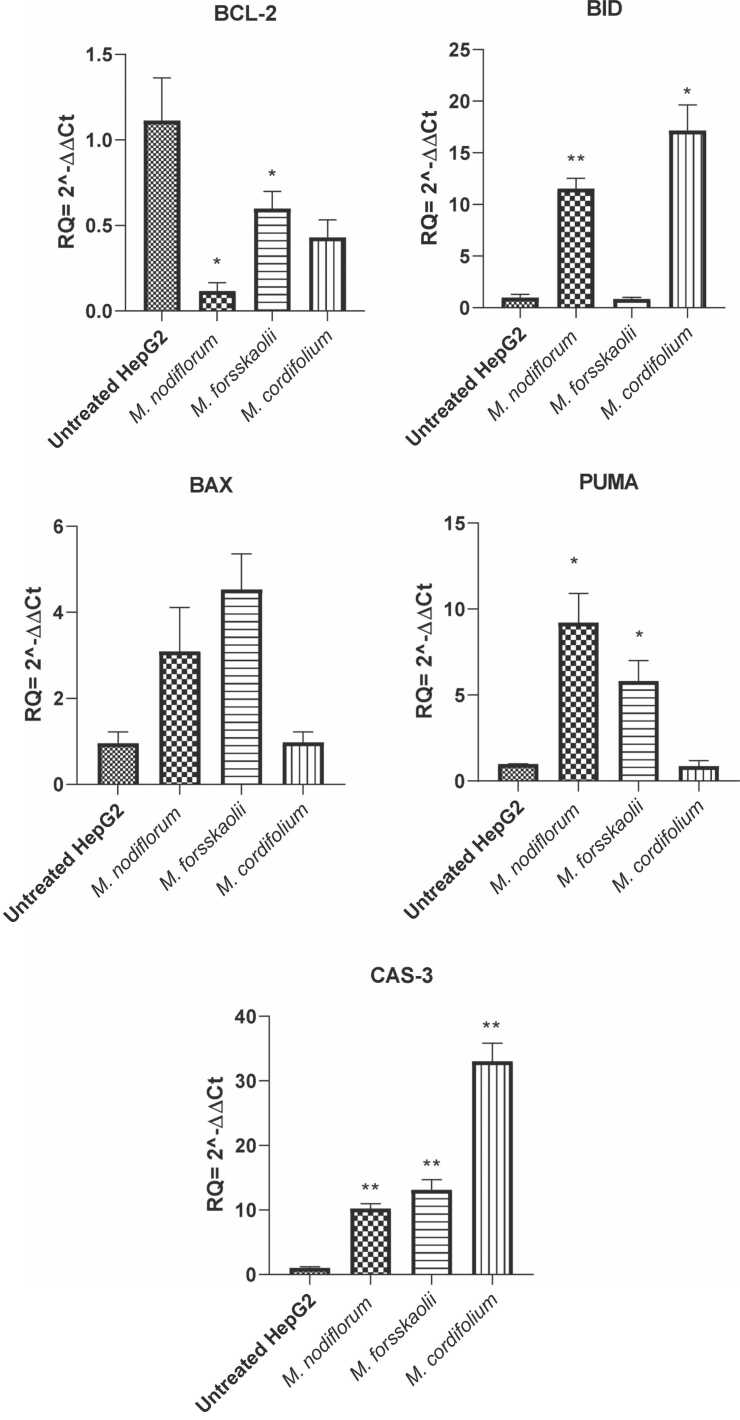
Apoptosis-related gene expression in HepG2 cells treated with different extracts, *M. nodiflorum*, *M. forsskaolii*, and *M. cordifolium.* Gene expression levels of BCL-2, BID, BAX, PUMA & Cas-3 in untreated HepG2 cells and treated HepG2 cells. Statistically significant differences from untreated cells are indicated by *p < 0.05 and **p < 0.01.

According to the results, only *M. frosskaolii* extract had a dual cytotoxic effect on both cell lines (CACO2 and HepG2), thus we further assessed molecular techniques on HepG2 cells. Anticancer activity of the three extracts on HepG2 cells was obvious thus further studies on apoptotic pathways were carried out to detect the exact mechanism of these extracts. Two-way ANOVA analysis showed *p*-value= 0.0048. Regarding the gene expression, the BCL-2 oncogenic marker revealed a slight decrease with *M. nodiflorum* treatment. While expression of PUMA in treatment with *M. cordifolium* was similar to that of the untreated cells. As for the CAS-3 enzyme, its level increased due to treatment with the three extracts, but it is maximum expression was observed in *M. cordifolium* Fig. 8. These molecular findings concluded that the three extracts influence the modulation of apoptosis in HepG2 cells in different ways which may involve different therapeutic relevance.

### 3.7 Detection of α-fetoprotein and LDH in treated HepG2 cells

**Fig. 9:**
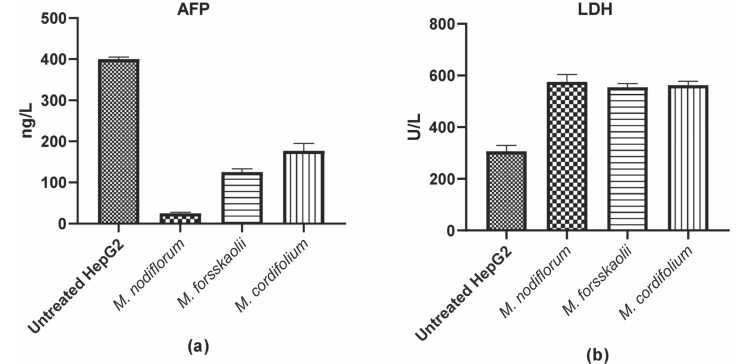
(a): Sandwich ELISA of α-fetoprotein in treated HepG2 cells compared to untreated. (b): LDH release detection in the treated HepG2 cells compared to untreated. *p*< 0.0001.

The ELISA measurements showed a considerable decrease in the concentration of AFP in HepG2 cells treated with extracts compared to untreated HepG2 cells. In untreated cells, AFP is about 400 ng/L which is considerably high however, treatment with extracts *M. nodiflorum*, *M. forsskaolii*, and *M. cordifolium* significantly decreases the AFP level. Nevertheless, *M. nodiflorum* displayed the remarked decline in AFP proving the inhibition of AFP synthesis in liver cancer cells. Fig. 9**a**

LDH detection in HepG2 denoting baseline cytotoxicity; 368 U/L. On the contrary, the LDH level in cells treated with *M. nodiflorum*, *M. forsskaolii,* and *M. cordifolium* showed a remarked increase with a range of 550–570 U/L, which indicates that the cytotoxicity of these samples is higher. This implies that all three extracts interfere with cell membranes. Fig. 9**b**

## 4 Discussion

*Mesembryanthemum* spp. is a genus of flowering plants belonging to the family Aizoaceae [32]. The genus includes several species where *Mesembryanthemum crystallinum* (known as the ice plant) and *Mesembryanthemum nodiflorum* are the most notable. Previous records on *Mesembryanthemum* spp. revealed significant biological activities particularly antibacterial and antioxidant properties [2], [20]. Further research could enhance the understanding and utilization of these plants in modern medicine. Our study aimed to determine the dominant compounds in the three extracts for an in-silico study on VEGFR as a receptor target. It also investigated the anti-inflammatory and anticancer properties of methanol extracts of the aerial parts of *M. nodiflorum*, *M. forsskaolii*, and *M. cordifolium*. Another computational analysis that helps to understand the biological effects of substances in different research situations was carried out by identifying the targets using SwissTargetPrediction. Firstly, we planned to study the cytotoxicity of the three plants’ extracts however, many previous studies of *Mesembryanthemum* spp. extracts of the same family were performed however the hemolytic effect was not the primary focus [33]. Thus, we emphasized the RBCs toxicity to assess the propensity of each extract to burst or lyse RBCs and thus release the hemoglobin in the surrounding solution. This is very important to define the safety of a compound since hemolysis at more than a certain level can point out adverse effects on human cells and potential toxicity in the case of therapeutic application [24], [34]. In addition, in vitro anti-inflammatory activity through the determination of protein inhibition percent exhibited that all tested extracts had anti-inflammatory activity. Kang and Joo exposed that the anti-inflammatory effect of the different organs (cotyledon, stem, and leaf) of the common ice plant *Mesembryanthemum crystallinum* L. was exhibited by inhibiting the production of inflammatory cytokines (TNF-α, IL-6, and IL-1β) [35]. Nevertheless, the anticancer results on CACO2 cells are compatible with data published in 2019 where treatment of HCT116 human colon cancer cells with ethanol extract and different solvent fractions of *M. crystallinum* L. resulted in dose-dependent inhibition of cell growth and induction of apoptosis through an increased G2/M cell population using flow cytometry [2]. A recent study in 2022 showed that *M. nodiflorum* had obvious anticancer activity on HepG2 cells with IC_50_ equals 1.39μ/well [33]. The GC-MS analysis identified dominant compounds in extracts from *M. nodiflorum*, *M. forsskaolii*, and *M. cordifolium* which are Hexadecanoic acid, 2-hydroxy-1-(hydroxymethyl) ethyl ester (Cpd-1), Benzeneethanamine, N,N-dimethyl- (Cpd-2), Octadecanoic acid, 2,3-dihydroxypropyl ester (Cpd-3), and 1H-indene, 1-hexadecyl-2,3-dihydro (Cpd-4). Computitional study using SwissTargetPrediction analysis exhibited that Cpd-1 targets kinases, oxidoreductases, and enzymes, while Cpd-2 acts on family A G protein-coupled receptors, oxidoreductases, and enzymes. Cpd-3 targets phosphorylation and enzymatic processes, while Cpd-4 targets nuclear receptors, family A G protein-coupled receptors, and membrane receptors. Only a previous study was performed using SwissTargetPrediction analysis on the alkaloidal fraction of *M. cordifolium* root proved to have an antidepressant effect even higher than imipramine hydrochloride, which is considered an antidepressant drug. Nine alkaloids were extracted from the metabolomic analysis concerning cancer diseases. Interestingly, among the dereplicated constituents, mesembrane (C_17_H_25_NO_2_) showed a strong tight, and specific interaction with SERT protein [36]. Consequently, our study focused on in silico comprehensive research to reinforce the medicinal value of *Mesembryanthemum* species. VEGFR works in anti-angiogenesis and turns out to be a suitable choice for halting the proliferation and metastasis of tumor cells. Most of the small synthetic molecule inhibitors up to date manifested good therapeutic efficacy in cancer treatment but they have side effects [37], [38]. Lv et al. studied the way that five old medications that target VEGFR2 interact with one another through computer simulations. Compared to sorafenib, five medications showed more consistent interactions with VEGFR2. The most stable binding affinity for VEGFR2 is shown by zafirlukast with a binding affinity equal to −10.9037 kcal/mol [39]. Herein, the results of docking studies showed specific characteristics of the interaction between the four major compounds and the VEGFR protein. To provide in-depth details on the specific mechanisms by which *Mesembryanthemum* spp. induce apoptosis, our study depicted a more targeted research to fully elucidate the apoptotic pathways in HepG2 modulated by *Mesembryanthemum* spp (*M. nodiflorum*, *M. forsskaolii*, and *M. cordifolium*) [2], [40]. Our results concluded that the expression of BID was significantly upregulated in *M. nodiflorum* and *M. cordifolium* treatment and a minimal upregulation in *M. forsskaolii* treatment. It was investigated that BID protein is downregulated in HCC when compared to normal liver tissues due to the imbalance between enhanced anti-apoptotic proteins and a reduced number of pro-apoptotic proteins that facilitate tumor cell proliferation [41], [42]. The expression of BAX noticeably increased with *M. nodiflorum* and showed an inconsistent pattern with both *M. forsskaolii* and *M. cordifolium* which was nearly equal to the expression level in the untreated cells. *M. nodiflorum* strongly promoted the expression of PUMA which induces apoptosis through the generation of both superoxide and hydrogen peroxide in a BAX-dependent manner [43], [44]. Monitoring AFP levels in the treated HepG2 cells with different extracts exhibited a decrease in concentration compared to untreated cells. Besides, LDH release due to cell membrane damage was obvious in the treated HepG2 cells which concludes the potential cytotoxicity of the tested extracts; *M. nodiflorum*, *M. forsskaolii,* and *M. cordifolium*. This LDH leakage assay enables the quantification of cytotoxicity and thus comparison between the treated and untreated cells to explain the function of the most promising potential therapeutic agent [45]. Overall, these data suggest that each extract can induce apoptosis by affecting the expression of specific genes, likely due to the varying compound compositions.

## 5 Conclusion

The comprehensive study of *Mesembryanthemum* spp. extracts showed significant biological effects, particularly as anti-inflammatory and anticancer activities. The GC-MS analysis revealed the highest number of phytoconstituents were found in the extracts of *M. forsskaolii* which exhibited promising anticancer activity compared to controls on CACO2 cells. On the other hand, the IC_50_ values for HepG2 cells proved that all three species had a noticeable perspective for targeting liver cancer as they could modulate apoptotic pathways through the upregulation of related genes. Moreover, the use of a computational analysis perspective in the in vitro research elevates the significance of these phytochemicals as potential therapeutic compounds. In general, this research brings out important knowledge regarding the nutraceutical prospect of *Mesembryanthemum* spp. Further studies will be required to conduct our conclusion with preclinical for confirming the potential therapeutic candidates of these extracts.

## Limitations and future perspectives

While the in vitro studies showed potent biological activity of both CACO2 and HepG2 cancer cell lines, future in vivo preclinical studies are required to validate the therapeutic properties and toxicity of these extracts in cancer treatment. Furthermore, although the computational-based prediction of the extracts against SwissTargetPrediction and molecular docking with VEGFR presents the molecular targets of the extracts, the in-silico study needs experimental biochemical analysis to explain the details of the interaction between the extracts and target proteins.

Thus, the phytochemical identification through GC-MS in this present study showed a range of bioactive compounds but further research using HPLC-MS/MS could elucidate a detailed phytochemical profiling of these extracts. Further studies about how these extracts affect the expression of genes and proteins, and the subsequent cancer-fighting properties of these plants will be valuable for a more comprehensive understanding of the extracts' pharmaceutical applications.

## Ethics approval and consent to participate

Ethical approval for the study was obtained from the TBRI Ethics Committee (FWA00010609; PT 841). This work did not involve human subjects, it did not require a clinical trial registration number. The research focused on evaluating the biological activity and safety profile in vitro. This research adhered to the guidelines outlined in the Declaration of Helsinki.

## Conflict of interest

The authors declared no potential conflicts of interest concerning the research, authorship, and/or publication of this article.

## Funding

The authors received no financial support for the research, authorship, and/or publication of this article.

## CRediT authorship contribution statement

**Eman Ahmed El-Wakil:** Writing – review & editing, Visualization, Validation, Supervision, Software, Resources, Project administration, Methodology, Investigation, Funding acquisition, Formal analysis, Data curation, Conceptualization. **Hend Okasha:** Writing – review & editing, Writing – original draft, Visualization, Validation, Supervision, Software, Resources, Project administration, Methodology, Investigation, Funding acquisition, Formal analysis, Data curation, Conceptualization. **Manal Mortady:** Writing – review & editing, Visualization, Validation, Supervision, Software, Resources, Project administration, Methodology, Investigation, Funding acquisition, Formal analysis, Data curation, Conceptualization. **Heba Raafat Mohamed:** Writing – review & editing, Writing – original draft, Visualization, Validation, Supervision, Software, Resources, Project administration, Methodology, Investigation, Funding acquisition, Formal analysis, Data curation, Conceptualization.

## Declaration of Competing Interest

The authors declare that they have no known competing financial interests or personal relationships that could have appeared to influence the work reported in this paper.

## References

[1] Rashdan H.R.M., Okasha H., Salem M.M., Abd El-Hady B.M., Ekram B. (2023). Investigation of novel HCV therapies: Boscia angustifalia & Boscia senegalensis extracts loaded on galactosylated chitosan nanoparticles synthesized by eco-friendly method for HCV treatment. Int. J. Biol. Macromol..

[2] Seo J.A., Ju J. (2019). Antioxidant and growth inhibitory activities of Mesembryanthemum crystallinum L. In HCT116 human colon cancer cells. J. Nutr. Heal..

[3] Botteri E., Iodice S., Bagnardi V., Raimondi S., Lowenfels A.B., Maisonneuve P. (2008). Smoking and colorectal cancer: A meta-analysis. JAMA.

[4] González-Garrido J.A., Gómez-García J.A., Hernández-Abreu O.I., Olivares-Corichi I.M., Pereyra-Vergara F., García-Sánchez J.R. (2024). Anticancer activity of sargassum fluitans extracts in different cancer cells. Anticancer. Agents Med. Chem..

[5] Okasha H. (2024). Fundamental uses of peptides as a new model in both treatment and diagnosis. Recent Pat. Biotechnol..

[6] Worm D.J., Els-Heindl S., Beck-Sickinger A.G. (2020). Targeting of peptide-binding receptors on cancer cells with peptide-drug conjugates. Pept. Sci..

[7] Taghizadeh M.S., Niazi A., Moghadam A., Afsharifar A. (2022). Experimental, molecular docking and molecular dynamic studies of natural products targeting overexpressed receptors in breast cancer. PLoS One.

[8] Noble R.L. (1990). The discovery of the vinca alkaloids - Chemotherapeutic agents against cancer. : Biochem. Cell Biol..

[9] Francis P.A., Kris M.G., Rigas J.R., Grant S.C., Miller V.A. (1995). Paclitaxel (Taxol) and Docetaxel (Taxotere): active chemotherapeutic agents in lung cancer. Lung Cancer.

[10] Wall M.E., Wani M.C. (1995). Camptothecin and taxol: discovery to clinic—thirteenth Bruce F. Cain memorial award lecture. Cancer Res.

[11] Krishnan K A., George Valavi S., Joy A. (2024). Identification of novel EGFR inhibitors for the targeted therapy of colorectal cancer using pharmacophore modelling, docking, molecular dynamic simulation and biological activity prediction. Anticancer. Agents Med. Chem..

[12] Shigeta K., Datta M., Hato T., Kitahara S., Chen I.X., Matsui A., Kikuchi H., Mamessier E., Aoki S., Ramjiawan R.R., Ochiai H., Bardeesy N., Huang P., Cobbold M., Zhu A.X., Jain R.K., Duda D.G. (2020). Dual programmed death receptor-1 and vascular endothelial growth factor receptor-2 blockade promotes vascular normalization and enhances antitumor immune responses in hepatocellular carcinoma. Hepatology.

[13] Ghasemali S., Barzegar A., Farajnia S., Rahmati M., Negahdari B., Etemadi A., Nazari A. (2023). VEGFR2 mimicking peptide inhibits the proliferation of human umbilical vein endothelial cells (Huvecs) by blocking VEGF. Anticancer. Agents Med. Chem..

[14] Okasha H., Samir S., Nasr S.M. (2021). Purified recombinant human Chromogranin A N46 peptide with remarkable anticancer effect on human colon cancer cells. Bioorg. Chem..

[15] Abdallah A.E., Mabrouk R.R., Al Ward M.M.S., Eissa S.I., Elkaeed E.B., Mehany A.B.M., Abo-Saif M.A., El-Feky O.A., Alesawy M.S., El-Zahabi M.A. (2022). Synthesis, biological evaluation, and molecular docking of new series of antitumor and apoptosis inducers designed as VEGFR-2 inhibitors. J. Enzym. Inhib. Med. Chem..

[16] Alanazi M.M., Eissa I.H., Alsaif N.A., Obaidullah A.J., Alanazi W.A., Alasmari A.F., Albassam H., Elkady H., Elwan A. (2021). Design, synthesis, docking, ADMET studies, and anticancer evaluation of new 3-methylquinoxaline derivatives as VEGFR-2 inhibitors and apoptosis inducers. J. Enzym. Inhib. Med. Chem..

[17] Abdallah A.E., Mabrouk R.R., Elnagar M.R., Farrag A.M., Kalaba M.H., Sharaf M.H., El-Fakharany E.M., Bakhotmah D.A., Elkaeed E.B., Al Ward M.M.S. (2022). New series of VEGFR-2 inhibitors and apoptosis enhancers: design, synthesis and biological evaluation. Drug Des. Devel. Ther..

[18] Abdelnaby R.M., El-Malah A.A., Fakhreldeen R.R., Saeed M.M., Nadeem R.I., Younis N.S., Abdel-Rahman H.M., El-Dydamony N.M. (2022). In vitro anticancer activity screening of novel fused thiophene derivatives as VEGFR-2/AKT dual inhibitors and apoptosis inducers. Pharmaceuticals.

[19] Ameer M.R., Khalid Z.M., Shinwari M.Ibrar, Ali H. (2021). Correlation among antidiabetic potential, biochemical parameters and gc-ms analysis of the crude extracts of Justicia adhatoda L. Pak. J. Bot..

[20] El-Amier Y.A., Alghanem S.M., Al-Hadithy O.N., Fahmy A.A., El-Zayat M.M. (2021). Phytochemical analysis and biological activities of three wild mesembryanthemum species growing in heterogeneous habitats. J. Phytol..

[21] Morsi E.A., Ahmed H.O., Abdel-Hady H., El-Sayed M., Shemis M.A. (2020). GC-analysis, and antioxidant, anti-inflammatory, and anticancer activities of some extracts and fractions of Linum usitatissimum. Curr. Bioact. Compd..

[22] Okasha H., Abdel-Hady H., Morsi E.A., El-Wakil E.A., Shemis M.A. (2022). In vitro cytotoxic activity and identification of bioactive compounds isolated from Olea europaea and Syzygium aromaticum Plants. Pharm. Chem. J..

[23] Okasha H., Dahroug H., Gouda A.E., Shemis M.A. (2023). A novel antibacterial approach of Cecropin-B peptide loaded on chitosan nanoparticles against MDR Klebsiella pneumoniae isolates. Amino Acids.

[24] Rashdan H.R.M., Okasha H., Abdelhakeem M.A., Mosallam A.M., Temairk H., Alhamzani A.G., Abou-Krisha M.M., Yousef T.A., Abdelmonsef A.H. (2022). Synthesis and in-vitro biological analyses of new quinazolin-2,4-dionederivatives. Egypt. J. Chem..

[25] Bugnon M., Röhrig U.F., Goullieux M., Perez M.A.S., Daina A., Michielin O., Zoete V. (2024). SwissDock 2024: major enhancements for small-molecule docking with attracting cavities and autodock vina. Nucleic Acids Res.

[26] Saber M.A., Okasha H., Khorshed F., Samir S. (2021). A novel cell-based in vitro assay for antiviral activity of interferons α, β, and γ by qPCR of MxA gene expression. Recent Pat. Biotechnol..

[27] Eimani B.G., Sanati M.H., Houshmand M., Ataei M., Akbarian F., Shakhssalim N. (2014). Expression and prognostic significance of Bcl-2 and Bax in the progression and clinical outcome of transitional bladder cell carcinoma. Cell J..

[28] Khoshsirat S., Abbaszadeh H.A., Khoramgah M.S., Darabi S., Mansouri V., Ahmady-Roozbahany N., Ahrabi B., Bahrami M., Vafaei-Nezhad S., Tahmasebinia F., Hassan M.P. (2019). Protective effect of photobiomodulation therapy and bone marrow stromal stem cells conditioned media on pheochromocytoma cell line 12 against oxidative stress induced by hydrogen peroxide. J. Lasers Med. Sci..

[29] Yuan Y.-G., Zhang S., Hwang J.-Y., Kong I.-K. (2018). Silver nanoparticles potentiates cytotoxicity and apoptotic potential of camptothecin in human cervical cancer cells. Oxid. Med. Cell. Longev..

[30] Dirican E., Özcan H., Karabulut Uzunçakmak S., Takım U. (2023). Evaluation expression of the Caspase-3 and Caspase-9 apoptotic genes in schizophrenia patients. Clin. Psychopharmacol. Neurosci..

[31] Kamiyama T., Takahashi M., Nakagawa T., Nakanishi K., Kamachi H., Suzuki T., Shimamura T., Taniguchi M., Ozaki M., Matsushita M., Furukawa H., Todo S. (2006). AFP mRNA detected in bone marrow by real-time quantitative RT-PCR analysis predicts survival and recurrence after curative hepatectomy for hepatocellular carcinoma. Ann. Surg..

[32] Klak C., Bruyns P.V. (2013). A new infrageneric classification for Mesembryanthemum (Aizoaceae: Mesembryanthemoideae). Bothalia.

[33] Elhawary S., Hassan M.H.A., Mostafa D., AbouZid S., Sleem A.A., Mohammed R. (2020). Comparative phytochemical and biological study for mesembryanthemum nodiflorum and Aptenia cordifolia plants growing in Egypt. Egypt. J. Chem..

[34] Farag M.R., Alagawany M. (2018). Erythrocytes as a biological model for screening of xenobiotics toxicity. Chem. Biol. Interact..

[35] Kang Y.W., Joo N.M. (2023). Comparative analysis on phytochemical properties, anti-oxidative, and anti-inflammatory activities of the different organs of the common ice plant Mesembryanthemum crystallinum L. Appl. Sci..

[36] Said A.A.E., Ali T.F.S., Attia E.Z., Ahmed A.S.F., Shehata A.H., Abdelmohsen U.R., Fouad M.A. (2021). Antidepressant potential of Mesembryanthemum cordifolium roots assisted by metabolomic analysis and virtual screening. Nat. Prod. Res..

[37] Yang J., Yan J., Liu B. (2018). Targeting VEGF/VEGFR to modulate antitumor immunity. Front. Immunol..

[38] Liu D., Ma X., Xiao D., Jia Y., Wang Y. (2018). Efficacy and safety of targeting VEGFR drugs in treatment for advanced or metastatic gastric cancer: a systemic review and metaanalysis. Oncotarget.

[39] Lv Y., Wang Y., Zheng X., Liang G. (2020). Reveal the interaction mechanism of five old drugs targeting VEGFR2 through computational simulations. J. Mol. Graph. Model..

[40] Arena R., Manuguerra S., Collins E., Mahdhi A., Renda G., Messina C.M., Santulli A. (2020). Antioxidant properties of a supercritical fluid extract of the halophyte Mesembryanthemum nodiflorum L. from sicilian coasts: Nutraceutical and cosmeceutical applications. Appl. Sci..

[41] Chen G.G., Lai P.B.S., Chan P.K.S., Chak E.C.W., Yip J.H.Y., Ho R.L.K., Leung B.C.S., Lau W.Y. (2001). Decreased expression of Bid in human hepatocellular carcinoma is related to hepatitis B virus X protein. Eur. J. Cancer.

[42] Luo Y.-X., Peng B.-Y., Chen Z.-H., Xiong X.-K., Huang J.-M., Chen M.-F., Wang F.-Y., Li X., Wang J.-N. (2022). The combination of chrysin and cisplatin induces apoptosis in HepG2 through down-regulation of cFLIP and activity of caspase. Anticancer. Agents Med. Chem..

[43] Zainodini N., Hajizadeh M.R., Mirzaei M.R. (2021). Evaluation of apoptotic gene expression in hepatoma cell line (HepG2) following nisin treatment. Asian Pac. J. Cancer Prev..

[44] Zhao T., Zhao C., Lu Y., Lin J., Tian Y., Ma Y., Li J., Zhang H., Yan W., Jiao P., Ma J. (2022). Noxa and Puma genes regulated by hTERT promoter can mitigate growth and induce apoptosis in hepatocellular carcinoma mouse model. J. Cancer.

[45] Liang W.F., Gong Y.X., Li H.F., Sun F.L., Li W.L., Chen D.Q., Xie D.P., Ren C.X., Guo X.Y., Wang Z.Y., Kwon T., Sun H.N. (2021). Curcumin activates ROS signaling to promote pyroptosis in hepatocellular carcinoma HepG2 cells. Vivo (Brooklyn).

